# Electro-haptic enhancement of speech-in-noise performance in cochlear implant users

**DOI:** 10.1038/s41598-019-47718-z

**Published:** 2019-08-06

**Authors:** Mark D. Fletcher, Amatullah Hadeedi, Tobias Goehring, Sean R. Mills

**Affiliations:** 1 Faculty of Engineering and Physical Sciences, University of Southampton, University Road, Southampton, SO17 1BJ United Kingdom; 2 MRC Cognition and Brain Sciences Unit, University of Cambridge, 15 Chaucer Road, Cambridge, CB2 7EF United Kingdom; 3 University of Southampton Auditory Implant Service, University of Southampton, University Road, Southampton, S017 1BJ United Kingdom

**Keywords:** Sensory processing, Human behaviour, Translational research

## Abstract

Cochlear implant (CI) users receive only limited sound information through their implant, which means that they struggle to understand speech in noisy environments. Recent work has suggested that combining the electrical signal from the CI with a haptic signal that provides crucial missing sound information (“electro-haptic stimulation”; EHS) could improve speech-in-noise performance. The aim of the current study was to test whether EHS could enhance speech-in-noise performance in CI users using: (1) a tactile signal derived using an algorithm that could be applied in real time, (2) a stimulation site appropriate for a real-world application, and (3) a tactile signal that could readily be produced by a compact, portable device. We measured speech intelligibility in multi-talker noise with and without vibro-tactile stimulation of the wrist in CI users, before and after a short training regime. No effect of EHS was found before training, but after training EHS was found to improve the number of words correctly identified by an average of 8.3%-points, with some users improving by more than 20%-points. Our approach could offer an inexpensive and non-invasive means of improving speech-in-noise performance in CI users.

## Introduction

When a sense is impaired or abolished, the brain adapts, relying more heavily on other senses to extract information^1,2^. This cross-modal plasticity could be exploited to enhance listening in deaf and hearing-impaired individuals fitted with a cochlear implant (CI). A CI is a neural prosthesis that bypasses the damaged or defunct outer and inner ear and electrically stimulates auditory nerve fibres to restore hearing. While, in normal-hearing individuals, speech information is transmitted to the brain by thousands of hair cells, in CI users it is transmitted through, at most, just 22 micro-electrodes. Because of this, much of the key information used to extract speech from a noisy background is not available. CI users therefore struggle to understand speech-in-noise much more than normal-hearing listeners^3,4^. Results from previous studies suggest that speech amplitude envelope information is a crucial missing feature for CI users listening in noise^5,6^. We hypothesize that, given the brain’s ability to extract missing information through another sense, speech-in-noise performance in CI users may be improved by providing speech envelope information through tactile stimulation. This haptic augmentation of the electrical signal from the CI will be referred to as “electro-haptic stimulation” (EHS).

Several studies have shown examples of sensory substitution, where information no longer transmitted through one sense is instead transmitted through another^7–10^. For example, researchers have shown that tactile aids (with no concurrent audio signal) can be used to transmit complex speech information^11–13^. Similarly, when a sense is impaired and therefore all of the critical information cannot be transferred, missing information can be provided through another sense^2,14,15^. Two recent studies showed examples of this sensory augmentation for CI listening^16,17^. Fletcher *et al*.^16^ found that tactile presentation of speech envelope improved speech intelligibility for normal-hearing subjects listening to CI simulations (NHCIs)^16^. Fletcher *et al*. extracted their tactile signal from speech-in-noise and presented it to the index finger. Huang *et al*. found similar results for CI listening in noise when the fundamental frequency was presented to the finger^17^, although in their study the tactile signal was extracted from the clean speech, rather than from the speech-in-noise. In the current study, we used an approach that is suitable for a real-world application. Three key features of our approach were: (1) as in Fletcher *et al*., we extracted the speech envelope from the speech-in-noise signal using a signal processing approach that was computationally lightweight and appropriate for real-time processing; (2) we delivered tactile stimulation to the wrist rather than the finger, which is a more suitable site for real-world use; and (3) we used a vibration intensity that can be produced by a near-silent, inexpensive, compact device with low power consumption. The aim of the current study was to establish whether our approach can be used to improve speech-in-noise performance in CI users.

It is likely that a short training regime will increase the benefit of EHS to speech-in-noise performance. Fletcher *et al*.^16^ found that training was important; the benefit of tactile stimulation to speech recognition in noise was found to grow substantially after just 30 minutes of exposure to speech-in-noise and concurrent tactile stimulation. Fletcher *et al*. used NHCIs whereas, in the current study, CI users were tested. Evidence that CI users integrate auditory and lip-reading cues more effectively than NHCIs has been used to suggest that CI users are better at integrating multisensory information than normal-hearing listeners^18^. It may therefore be expected that CI users will benefit more from tactile stimulation. However, it is possible that lip-reading represents a special case as lip-reading cues are often used to aid speech understanding in real-world situations, whereas speech envelope information is never presented through touch in real-world settings. The novelty of the tactile stimulus could be distracting at first or less readily integrated with auditory information. It is therefore possible that training or familiarisation will be required to facilitate the multisensory integration needed for CI users to benefit from EHS.

To investigate the effect of EHS, 10 CI users completed a speech testing session, followed by two training sessions, and then a final testing session. In the testing sessions, participants completed a speech-in-noise task, where the percentage of correctly identified keywords in noise was measured both with and without concurrent tactile stimulation of the wrists. Speech testing was conducted with multi-talker noise, in which CI users are known to struggle most^19,20^. In the training sessions, participants received speech-in-noise training with concurrent tactile stimulation. These training sessions used audiobook material with different talkers from the one used in the testing sessions. The two training sessions totalled just 20 minutes of exposure to speech-in-noise and tactile stimulation. It is anticipated that, after training, EHS will increase the percentage of keywords in noise that CI users are able to correctly identify.

## Results

**Figure 1:**
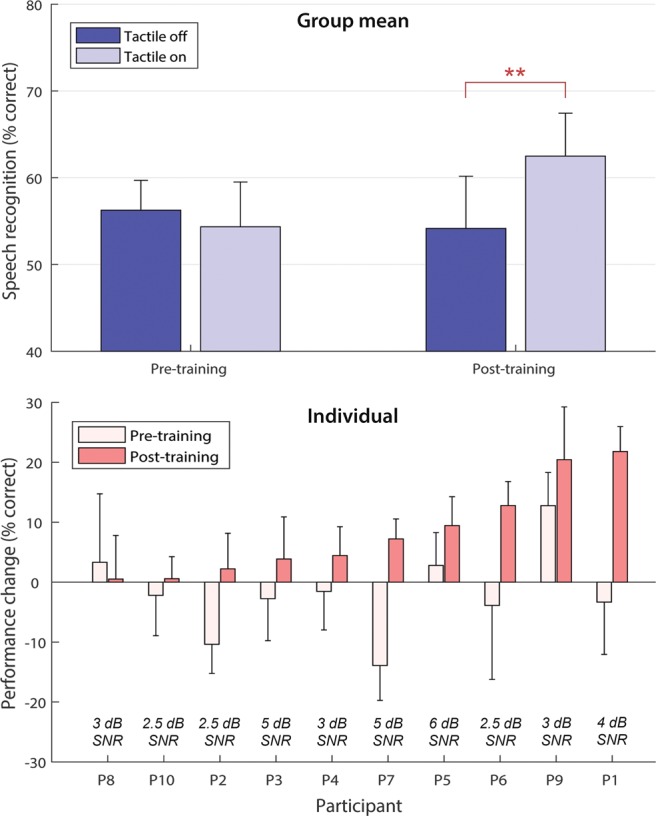
The top panel shows the mean speech-in-noise performance across all participants with and without tactile stimulation, both before and after training. The bottom panel shows the difference in performance with and without tactile stimulation for each individual, before and after training. A positive performance change indicates that performance was better with tactile stimulation. Participants are ordered by the size of their post-training performance change. The signal-to-noise ratio (SNR) at which speech-in-noise performance was measured for each individual is shown in the bottom panel. Error bars show the standard error of the mean (SE).

Figure 1 shows the effect of tactile stimulation on speech-in-noise performance (the percentage of key words correctly identified) before and after training. The results were analyzed using a repeated-measures ANOVA, with factors ‘Session’ (before or after training) and ‘Condition’ (with or without tactile stimulation). There was a significant interaction effect, indicating that the effect of tactile stimulation in the post-training session was significantly larger than in the pre-training session (*F*(1,9) = 14.2, *p* = 0.004, *η*_*p*_^2^ = 0.917). Main effects of Condition and Session were non-significant.

Paired t-tests (with a Bonferroni corrected alpha of 0.025), revealed a significant effect of Condition in the post-training session (*t*(9) = 3.4, *p* = 0.008, *d* = 1.08), but not in the pre-training session (*t*(9) = 0.82, *p* = 0.43). Before training, in 7 out of 10 participants’ performance was worse with tactile stimulation, and individual results were highly variable. The largest improvement in performance with tactile stimulation before training was 12.8%-points (improving from 54.5% to 67.2%, *SE* = 5.5, P9), and the largest reduction in performance was 13.9%-points (decreasing from 36.1% to 22.2%, *SE* = 5.8, P7). There was also substantial variability within participants, with the difference in performance across sentence lists within a single session and condition being as much as 44.4%-points (the condition with tactile stimulation, session 1 for P6). After training, all ten participants correctly identified more keywords with tactile stimulation, with the smallest improvement being 0.5%-points (improving from 67.8% to 68.3%; *SE* = 7.3%, P8) and the largest improvement being 21.8%-points (33.8% to 55.6%, *SE* = 4.2%, P1). The mean improvement with tactile stimulation after training was 8.3%-points (improving from 54.3% to 62.5%; *SE* = 2.5%). It should be noted that, while the order of the sentence lists used for the two conditions (with and without tactile stimulation) was counterbalanced across our 10 participants, the sentence lists used were not fully counterbalanced between the two sessions (before and after training).

## Discussion

EHS was found to significantly improve speech-in-noise performance after a short training regime. The regime consisted of just 20 minutes of exposure to speech-in-noise material with concurrent tactile stimulation. Tactile stimulation increased the number of words correctly identified by 8.3%-points on average compared to audio alone, with some individuals improving by more than 20%-points and no individuals decreasing in performance. This benefit was observed for speech in multi-talker noise, where CI users are known to struggle most^19,20^, and in which noise reduction algorithms typically perform poorly^21,22^. The tactile signal was delivered at an intensity that could readily be produced by a low-cost wearable device after computationally non-intensive signal processing suitable for a real-time application.

Fletcher *et al*.^16^, who presented speech envelope through tactile stimulation to the index finger of NHCIs, found a slightly larger benefit of tactile stimulation for speech-in-noise performance than in the current study (10.8%-points compared to 8.3%-points, on average). The current study deployed tactile stimulation to the wrist, rather than the fingertip. The wrist, while being a more practical site for real-world use, has higher vibro-tactile thresholds than the fingertip^23^ and a lower density of mechanoreceptors^24^. However, researchers have shown better perception of amplitude differences at the wrist than at the fingertip as well as similar gap detection and discrimination of frequency differences^23^. It is therefore possible that the wrist could be similarly capable of transferring speech information. Another difference between the studies was that the current study was conducted in CI users who were older than the NHCIs tested by Fletcher *et al*. (61.2 years on average compared to 25.5 years). Tactile sensitivity is known to be reduced in older adults^25,26^ and age also affects integration of information from more than one sense^27^. These limitations may reduce the benefit of EHS in older CI users. Finally, there was less training (20 minutes compared to 30 minutes) in the current study. Training has been found to be highly important in previous work in tactile aids (with no concurrent audio signal^11^) and large training effects were found both in the current study and in Fletcher *et al*. It therefore seems likely that the smaller amount of training used here may have led to a smaller benefit from tactile stimulation. Another study, by Huang *et al*.^17^, reported robust benefits to speech-in-noise performance from EHS without training when the fundamental frequency of speech was derived from the clean speech signal and presented to the fingertip. However, the size of the benefit is difficult to compare directly to the current results because of the different outcome measures and speech corpora used.

There was a great deal of variation in the size of the EHS benefit between individuals, with the improvement in performance after training ranging from 0.5 to 21.8%-points. It is important for future research to establish which factors are most important in determining how much a CI user will benefit from EHS. For example, it should be established whether EHS can provide benefit in congenitally deaf individuals and those who became deaf prelingually. Interestingly, there is evidence of enhanced tactile sensitivity in congenitally deaf individuals^28^ and that congenitally deaf individuals are able to effectively integrate audio and tactile information^29^, suggesting that this group may benefit from EHS. It will also be important to establish which factors influence how much training is needed for EHS to be effective. Before training, there was large variability in the effect of EHS, with most participants’ performance decreasing with tactile stimulation (by more than 10%-points in two participants; P2 and P7), and one participant’s performance improving by 12.8%-points (P9). Both of the participants who had substantial decreases in performance with tactile stimulation before training reported to the experimenter that they found tactile stimulation distracting in the pre-training session. Intriguingly, the participant who improved with tactile stimulation before training had previously learned to play the flute using vibration from the instrument when they had a severe hearing impairment and before they were implanted. This may suggest that learning to use tactile cues for auditory stimuli can generalize from one context (in this case music) to another (in this case speech) and that the ability to use tactile cues can be retained over many years.

In the current study, training consisted of just 20 minutes of exposure to speech-in-noise and concurrent tactile stimulation over the course of two one-hour sessions, and no exposure to speech-in-noise alone. It is possible that the absence of training with speech-in-noise alone created a bias in the post-training testing session towards the condition with EHS. Training without tactile stimulation was not included as an increase in number of visits, a substantial increase in the duration of each visit, or a decrease in the already small amount of training with tactile stimulation would have been required. An increase in the number of visits would have made recruitment of participants, who often live a significant distance from the research centre, extremely difficult. An increase in the one-hour session time was not deemed appropriate as participants reported finding the training very tiring. Biasing towards the condition with EHS was unlikely to have occurred for three main reasons: (1) participants “train” extensively on speech-in-noise without tactile stimulation in their everyday life outside of the laboratory sessions using the same CI settings as in the experiment; (2) participants trained with different talkers and different material from in the testing sessions, and so were unable to generate a content- or talker-specific bias for EHS; and (3) participant expectation that EHS would be beneficial was controlled by falsely instructing the participants that the experiment compared “audio enhancement” with “vibration enhancement”. This instruction was reinforced in each trial in the testing sessions and the experimenter received no indication that participants were sceptical about the truth of this statement. Furthermore, the experimenter remained blinded to whether EHS was being provided throughout the testing sessions to prevent experimenter bias. In future work, we plan to develop a remote training programme to increase the training time available and thereby allow for further control of bias effects.

As well as allowing further experimental control, the development of a remote training programme would allow several important extensions of the current work. For example, it will be important to establish which training methods provide the most benefit of EHS over long time periods in a real-world setting. The development of interactive, game-like training software with clear goals and rewards, which maximizes user motivation and enjoyment and minimizes the effort required to train, is likely to be important for ensuring maximal uptake of EHS. Once an effective long-term training regime has been established, it will also be highly interesting to investigate whether EHS can be used to accelerate patient acclimatization to a new CI and whether some of the benefits of EHS remain even when tactile stimulation is no longer provided. Finally, as future work moves EHS closer to a real-world application, it will be important to establish the practicality of using a wearable haptic device, exploring, for example, the robustness of EHS to gesticulation and other arm movements.

Future research could also explore whether EHS can enhance spatial hearing. Spatial hearing is severely impaired in CI users, particularly in those with only one implant^30,31^, who make up 93% of users in the UK^32^. As well as allowing CI users to better locate auditory objects in space, improved spatial hearing might increase the release from masking that occurs when speech is in a separate spatial location to a masking sound^33–35^. Remarkably, it has been shown that, using only tactile stimulation, subjects are able to discriminate the location of concurrent sounds whose direction of origin is separated by less than 5°^36^. Furthermore, other research suggests that the tactile system can make use of tactile intensity differences that originate from sounds whose intensities differ by as little as 1 dB^37^. Such a difference in sound level between the ears (interaural level difference) would correspond to a horizontal angle change of just a few degrees for sounds with frequency content above ~1 kHz^38^. The current study was conducted with tactile stimulation to both wrists, a set up that could easily be adapted to provide interaural level difference cues to improve spatial hearing in CI users.

## Materials and Methods

### Participants

Ten CI users (4 male, 6 female; mean age = 61.2 years, ranging from 41 to 70 years) were recruited through the University of Southampton Auditory Implant Service. Participants were only invited to take part in the experiment if they were: (1) native British English speakers, (2) aged between 18 to 70 years old (inclusive), (3) had been implanted at least 12 months prior to the experiment, (4) had no EAS (i.e. no functional residual low-frequency hearing; >65 dB HL at 250 and 500 Hz), (5) had speech in quiet scores of at least 70% (for Bamford-Kowal-Bench sentences; BKB) measured at their most recent clinical appointment, and (6) had the capacity to give informed consent. Table 1 details participant characteristics. All participants also reported on a screening questionnaire (see Fletcher *et al*.^16^) that they had no medical conditions and were taking no medication that might affect their touch perception. Participants gave written informed consent and received an inconvenience allowance.

**Table 1:** Summary of participant information.

Participant	Gender	Age	Speech-in-quiet	Type of implant	Time since implantation	Dominant hand
		(years)	(% correct)		(years)	
1	F	65	85	MEDEL Sonata	9.3	Right
2	M	56	89	AB Hi Res 90 k	3.4	Right
3	M	69	99	AB Hi Res 90 k	1.5	Right
4	F	68	98	Cochlear Nucleus 512 (CA) profile	3.7	Left
5	M	68	94	AB HiRes ultra	1.0	Right
6	F	70	96	Cochlear Nucleus 512 (CA) profile	2.7	Right
7	M	70	92	Cochlear Nucleus Freedom contour	9.1	Right
8	F	44	98	Cochlear nucleus 512 (CA) profile	1.6	Right
9	F	41	93	Cochlear nucleus contour (bilaterally)	7.7	Right
10	F	52	100	AB Hi Res 90 k	10.9	Right
*Mean*		*60.3*	*94.4*		*5.1*	

Vibro-tactile detection thresholds were determined for both the left and right index fingers for sinusoidal vibrations of 31.5 and 125 Hz, using conditions specified in ISO 13091-1:2001. Two participants (P2 and P3) had higher than normal thresholds (>0.7 ms^−2^ root mean square [RMS]^39^) at 125 Hz on the index finger of their left hand, and two other participants (P1 and P7) had elevated thresholds at 125 Hz on the index finger of their right hand. All other measured thresholds at the index finger were at normal levels.

### Tactile signal processing

**Figure 2:**
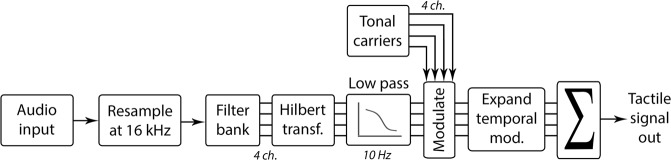
Schematic representation of the signal processing chain for the tactile signal generation.

Figure 2 shows a schematic representation of the signal processing chain that was used to convert the audio signal to a tactile signal. The signal processing approach is described in detail in Fletcher *et al*.^16^ and is summarized below. The parameters used in the current study were the same as in Fletcher *et al*., except that a smaller number of tonal carriers were used over a reduced frequency range. The reduced frequency range of the tactile signal (50–230 Hz) means that it can be more easily produced by a low-power wearable device that is near-silent.

**Figure 3:**
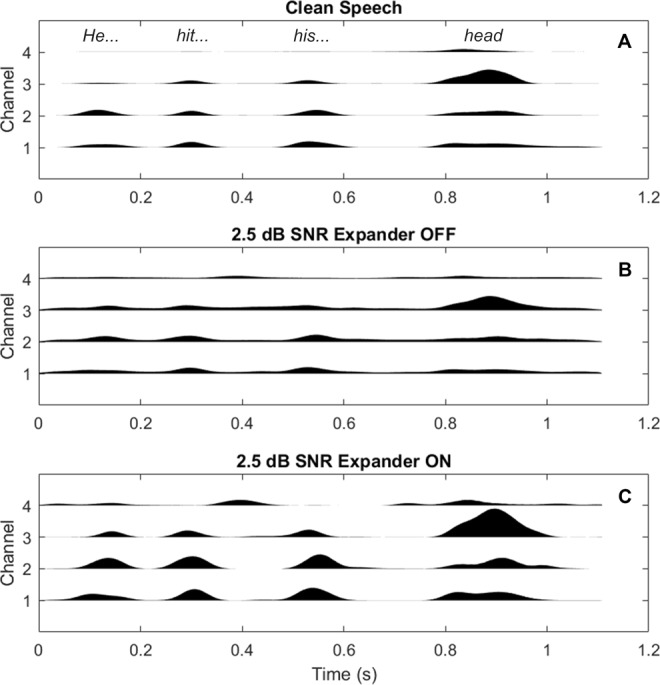
Illustration of the effect of the expander on the tactile signal amplitude for the sentence “He hit his head”. Panels A and B show the tactile signal for clean speech and for speech-in-noise (at 2.5 dB, the lowest SNR used in the study). Panel C shows the same signal as panel B, but with the expander on. The height of each channel waveform corresponds to the amplitude of the signal.

To generate the tactile signal, the audio input signal was first downsampled to a sampling frequency of 16 kHz. The signal was then passed through an FIR filter bank with 4 channels that were equally spaced on the ERB scale with upper and lower centre frequencies of 100 and 1000 Hz. The Hilbert envelope for each channel was computed and a first-order low-pass filter was applied with a cut-off frequency of 10 Hz. The channels were then used to modulate the amplitude envelopes of four fixed-phase tonal carriers with center frequencies of 50, 110, 170, and 230 Hz (a frequency range in which the tactile system is highly sensitive^40^). Each of these carrier signals was individually passed through an expander function (as used in Fletcher *et al*.) to amplify temporal modulations and reduce the contribution from the background noise, thereby increasing the salience of speech envelope information. Figure 3 illustrates the effect of the expander by showing the same sentence as: processed clean speech without the expander (panel A), processed speech-in-noise at an SNR of 2.5 dB with the expander turned off (panel B), and the same speech-in-noise signal with the expander turned on (panel C). The enhanced tonal carriers were then summed to form the input signal for tactile presentation to the participant. A 10-ms delay was added, to keep the tactile signal approximately synchronized with the electrical signal from the CI. The tactile signal was presented through two *HVLab* tactile vibrometers. The mean acceleration magnitude of the vibration output for a single sentence was 1.84 ms^−2^ RMS.

### Speech and noise stimuli

The speech signal was presented at a level of 65 dB SPL LAeq. Two different speech corpora were used in this study. The BKB Institute of Hearing Research male sentence corpus^41^ was used for speech testing. This material was not familiar to participants as clinical audiology test procedures use a different BKB sentence corpus. Training was conducted using speech material from the RealSpeech^TM^ (UK) content library (used with permission of Dr Ian Wiggins and Dr Mark Fletcher), which used two talkers (one male and one female) that are different from the talker used in the BKB sentence corpus. RealSpeech material comprises a set of narratives that cover a variety of general-interest topics recorded under near-anechoic conditions. A non-stationary multi-talker noise recorded by the National Acoustic Laboratories (NAL)^42^ was used in both training and speech testing. The noise was recorded at a party and had a spectrum that matched the international long-term average speech spectrum^43^.

### Apparatus

Participants were seated in a sound-attenuated booth with a background noise level conforming to the 2017 British Society of Audiology recommendations^44^. All stimuli were generated and controlled using custom MATLAB scripts (version R2016a, The MathWorks Inc, Natick, MA, USA). Acoustic stimuli were generated by a laptop located in a separate observation room and played out via an RME Babyface Pro soundcard (Haimhousen, Germany; sample rate of 44.1 kHz and bit depth of 24 bits) and a Genelec 8020 C PM Bi-Amplified Monitor System positioned at head height 1.5 m from the participant. The acoustic stimuli were calibrated at the listening position using a Brüel & Kjær (B&K) G4 type 2250 sound level meter and B&K type 4231 sound calibrator.

Two *HVLab* tactile vibrometers were placed beside the participant’s chair and were used to deliver the vibro-tactile signal to the participants’ wrists. The vibrometers in this experiment were adapted by the substitution of the standard 6-mm probe with a 10-mm probe, and the removal of the rigid surround. These changes increased the area of skin excitation. A B&K type 4294 calibration exciter was used to calibrate the accelerometers, and the vibration signal was calibrated to provide equal amplitude across the frequency range. A further *HVLab* tactile vibrometer with a 6-mm probe and a rigid surround was used to measure vibro-tactile detection thresholds using *HVLab* diagnostic software.

During testing, the experimenter sat in the control room with no line of sight to the participant. The sentences spoken by the participants were captured using a Shure BG 2.1 dynamic microphone, amplified by a GSI-61 audiometer, and presented to the experimenter through the GSI-61 Operator’s Headset.

### Procedure

**Figure 4:**
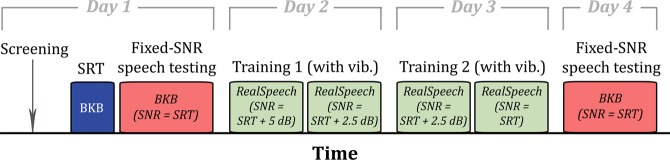
Schematic (not to scale) showing the timeline of the experiment. Speech material was either BKB sentences or audiobook material from RealSpeech.

Figure 4 shows a schematic illustration of the experimental procedure. Participants attended four sessions over a two-week period (mean period between first and last sessions = 5.4 days, ranging between 3 and 9 days, with an average gap between any two sessions of 1.8 days). The four sessions consisted of a speech testing session, followed by two training sessions, and then a final testing session. This procedure is similar to the one detailed in Fletcher *et al*.^16^, although here only 2 days of training were conducted, totalling around 20 minutes of exposure to speech in noise and concurrent tactile stimulation.

In the pre-training testing session, participants completed a screening questionnaire and had their vibro-tactile detection thresholds measured at the fingertip to check for normal touch perception. Detection thresholds were also measured for each wrist (where tactile stimulation was applied in this study) at 31.5 Hz (Mean = 0.79 ms^−2^ RMS) and 125 Hz (Mean = 0.68 ms^−2^ RMS). Each participant’s speech reception threshold (SRT) was then measured without tactile stimulation. Two SRT estimates were made, each using a single BKB sentence list (containing 15 sentences) mixed with multi-talker noise. Trials were marked as correct if participants correctly repeated at least 1 out of 3 keywords. For each SRT estimate, the SNR of the first trial was 5 dB. The sentence used in the first trial was then repeated, with the SNR increased by 2 dB after each repeat, until a trial was marked as correct. A one-up one-down adaptive tracking procedure^45^ with a step size of 2 dB was then followed for the remaining sentences for each SRT. The SRT was calculated as the mean of the last 4 reversals.

In both the pre- and post-training testing sessions, speech-in-noise performance was measured at an SNR equal to the mean of the two SRT estimates made in the first session. Speech testing was conducted with and without concurrent tactile stimulation. In both testing sessions, sentences were played to the participants and they were asked to repeat back what was said. Performance was measured as the proportion of key words that the participants correctly identified. The experimenter was able to use a text display to instruct participants to repeat their response if it was unclear. Sixteen BKB sentence lists were used in total, eight in each session. Half of the lists were played with concurrent tactile stimulation and half without. The two conditions were alternated in an A-B-A-B pattern across the lists and were counterbalanced across participants. The order of conditions was swapped between the pre- and post-training sessions (i.e. B-A-B-A). During the speech testing, participants were instructed via a text display to either place their wrists on the vibrometer contacts, or to place their arms on the armrests of the chair. The tactile signal was played through the vibrometers in both conditions. When participants had their wrists on the contacts, the message “Vibration enhancement ON. Audio enhancement OFF.” was displayed on the screen, and when they had their arms on the armrests the message “Vibration enhancement OFF. Audio enhancement ON.” was displayed. This latter message falsely stated that the audio signal had been enhanced in the condition without tactile stimulation. This deceptive cue was included to control for effects of participant expectation that tactile stimulation was intended to improve performance. The experimenter was blinded to which conditions contained tactile stimulation, in order to avoid experimenter bias.

The two training sessions consisted of target speech from the RealSpeech content library. In each session the participant listened to two speech segments, each lasting around five minutes. Half of the segments were read by a female talker and half by a male talker and were presented in a random order. The segments were split into single sentences and mixed with the NAL multi-talker noise. The first segment was presented at an SNR 5 dB above the participants SRT, the second and third at 2.5 dB above, and the final segment at an SNR equal to the participant’s SRT. Participants were asked to repeat each sentence to the experimenter after which the correct sentence text was displayed on the screen. For all training material concurrent tactile stimulation was provided.

The experimental protocol was approved by the University of Southampton Ethics Committee (ERGO ID: 30753) and the UK National Health Service Research Ethics Service (Integrated Research Application System ID: 244309). All research was performed in accordance with the relevant guidelines and regulations.

## Data Availability

The dataset from the current study is publicly available through the University of Southampton’s Research Data Management Repository: 10.5258/SOTON/D1034.
